# Temporal colonization and metabolic regulation of the gut microbiome in neonatal oxen at single nucleotide resolution

**DOI:** 10.1093/ismejo/wrad022

**Published:** 2024-01-10

**Authors:** Quanbin Dong, Dongxu Hua, Xiuchao Wang, Yuwen Jiao, Lu Liu, Qiufeng Deng, Tingting Wu, Huayiyang Zou, Chen Zhao, Chengkun Wang, Jiafa Reng, Luoyang Ding, Shixian Hu, Jing Shi, Yifeng Wang, Haifeng Zhang, Yanhui Sheng, Wei Sun, Yizhao Shen, Liming Tang, Xiangqing Kong, Lianmin Chen

**Affiliations:** Department of Cardiology, The First Affiliated Hospital of Nanjing Medical University, Nanjing Medical University, Nanjing 210029, China; Department of Cardiology, The First Affiliated Hospital of Nanjing Medical University, Nanjing Medical University, Nanjing 210029, China; Department of Cardiology, The First Affiliated Hospital of Nanjing Medical University, Nanjing Medical University, Nanjing 210029, China; Changzhou Medical Center, The Affiliated Changzhou No.2 People's Hospital of Nanjing Medical University, Nanjing Medical University, Changzhou 213164, China; Changzhou Medical Center, The Affiliated Changzhou No.2 People's Hospital of Nanjing Medical University, Nanjing Medical University, Changzhou 213164, China; Department of Cardiology, The First Affiliated Hospital of Nanjing Medical University, Nanjing Medical University, Nanjing 210029, China; Department of Cardiology, The First Affiliated Hospital of Nanjing Medical University, Nanjing Medical University, Nanjing 210029, China; Department of Cardiology, The First Affiliated Hospital of Nanjing Medical University, Nanjing Medical University, Nanjing 210029, China; Department of Cardiology, The First Affiliated Hospital of Nanjing Medical University, Nanjing Medical University, Nanjing 210029, China; Department of Cardiology, The First Affiliated Hospital of Nanjing Medical University, Nanjing Medical University, Nanjing 210029, China; Department of Cardiology, The First Affiliated Hospital of Nanjing Medical University, Nanjing Medical University, Nanjing 210029, China; Department of Cardiology, The First Affiliated Hospital of Nanjing Medical University, Nanjing Medical University, Nanjing 210029, China; College of Animal Science and Technology, Yangzhou University, Yangzhou 225009, China; Institute of Precision Medicine, The First Affiliated Hospital, Sun Yat-sen University, Guangzhou 510080, China; Department of Cardiology, The First Affiliated Hospital of Nanjing Medical University, Nanjing Medical University, Nanjing 210029, China; Cardiovascular Research Center, The Affiliated Suzhou Hospital of Nanjing Medical University, Gusu School, Nanjing Medical University, Suzhou Municipal Hospital, Suzhou 215006, China; Cardiovascular Research Center, The Affiliated Suzhou Hospital of Nanjing Medical University, Gusu School, Nanjing Medical University, Suzhou Municipal Hospital, Suzhou 215006, China; Cardiovascular Research Center, The Affiliated Suzhou Hospital of Nanjing Medical University, Gusu School, Nanjing Medical University, Suzhou Municipal Hospital, Suzhou 215006, China; Department of Cardiology, The First Affiliated Hospital of Nanjing Medical University, Nanjing Medical University, Nanjing 210029, China; College of Animal Science and Technology, Hebei Agricultural University, Baoding 071000, China; Changzhou Medical Center, The Affiliated Changzhou No.2 People's Hospital of Nanjing Medical University, Nanjing Medical University, Changzhou 213164, China; Department of Cardiology, The First Affiliated Hospital of Nanjing Medical University, Nanjing Medical University, Nanjing 210029, China; Cardiovascular Research Center, The Affiliated Suzhou Hospital of Nanjing Medical University, Gusu School, Nanjing Medical University, Suzhou Municipal Hospital, Suzhou 215006, China; Department of Cardiology, The First Affiliated Hospital of Nanjing Medical University, Nanjing Medical University, Nanjing 210029, China; Changzhou Medical Center, The Affiliated Changzhou No.2 People's Hospital of Nanjing Medical University, Nanjing Medical University, Changzhou 213164, China

**Keywords:** gut microbiome, metagenomics, single nucleotide variations, metabolomics

## Abstract

The colonization of microbes in the gut is key to establishing a healthy host-microbiome symbiosis for newborns. We longitudinally profiled the gut microbiome in a model consisting of 36 neonatal oxen from birth up to 2 months postpartum and carried out microbial transplantation to reshape their gut microbiome. Genomic reconstruction of deeply sequenced fecal samples resulted in a total of 3931 metagenomic-assembled genomes from 472 representative species, of which 184 were identified as new species when compared with existing databases of oxen. Single nucleotide level metagenomic profiling shows a rapid influx of microbes after birth, followed by dynamic shifts during the first few weeks of life. Microbial transplantation was found to reshape the genetic makeup of 33 metagenomic-assembled genomes (FDR < 0.05), mainly from *Prevotella* and *Bacteroides* species. We further linked over 20 million microbial single nucleotide variations to 736 plasma metabolites, which enabled us to characterize 24 study-wide significant associations (*P* < 4.4 × 10^−9^) that identify the potential microbial genetic regulation of host immune and neuro-related metabolites, including glutathione and L-dopa. Our integration analyses further revealed that microbial genetic variations may influence the health status and growth performance by modulating metabolites via structural regulation of their encoded proteins. For instance, we found that the albumin levels and total antioxidant capacity were correlated with L-dopa, which was determined by single nucleotide variations via structural regulations of metabolic enzymes. The current results indicate that temporal colonization and transplantation-driven strain replacement are crucial for newborn gut development, offering insights for enhancing newborn health and growth.

## Introduction

The colonization of the microbiome in neonatal animals is crucial, as these resident microbes support many functions, including the maturation of the immune system, the utilization and modification of nutrients, and the prevention of pathogen colonization [1–4]. Although dairy cattle harbor a diverse community of ruminal bacteria that provides energy for growth and milk production, the rumen of the neonatal calf is still under development and exhibits limited functionality during early life [5]. Therefore, the acquisition and colonization of the gut microbiome are key to establishing a healthy host-microbiome symbiosis in the neonatal calf. Thus, it is important to elucidate the temporal dynamics of the gut microbiome during early life to understand the relationships between the gut microbiome and the health status of neonatal oxen. Eventually, this will help in designing intervention strategies to achieve better health and growth performance.

Studies in neonatal oxen have demonstrated that the microbial taxonomic composition in hindgut changes dramatically after birth [6–8]. For example, within 24 h of giving birth, there is a significant increase in the abundances of *Enterobacteriaceae* and *Enterococcus* [6]. After a week of birth, *Lactobacillus*, *Faecalibacterium*, and mucosa-associated *Escherichia* are significantly more abundant [7–9]. The fecal microbiota of 3-week-old oxen was predominantly composed of *Bacteroides*, *Prevotella*, *Coccus-Useriella*, and *Faebacillus* [10], while at 5 weeks, it primarily consisted of *Lactococcus flavus* and cellulolytic bacteria. *Bacteroides prevotella*, *Clostridium coccoides*, and *Eubacterium rectale* constituted the major fraction of the microbiota within 12 weeks after birth [9]. By the age of 2 years, the bacterial composition was mainly dominated by *Coriobacteriales* order [11]. These studies collectively demonstrate that the gut microbiota undergoes age-related changes. Furthermore, the composition and structure of the intestinal microbiome can vary among individuals over time [12]. These observations have laid the foundation for targeted mechanistic investigations into the consequences of host-microbiome crosstalk for the health and growth of newborn oxen.

Investigations into the temporal dynamics of the gut microbiome of newborn oxen have mainly focused on the taxonomic and functional composition and have not explored how microbial strains evolve over time. Each microbial species consists of different strains that vary in single nucleotide variation (SNV) and may have different functions [13–16]. Recently, crucial links between microbial genetic composition and longevity [17], as well as multiple risk factors of disease [18, 19], have been revealed in humans. Therefore, tracking the temporal dynamics of microbial genetic variations and linking them with the health status and growth of newborn oxen may provide us with a new layer of information regarding the role of the gut microbiome in neonatal animals.

In this study, we conducted a longitudinal follow-up of the gut microbiome of 36 neonatal oxen from birth up to 2 months postpartum and performed microbial transplantation (MT) to evaluate whether altering microbial strains may influence the health status and growth performance of the oxen. We characterized the temporal stability of the gut microbial genomic makeup of the oxen, and further investigated the link between temporal variations and the health status and growth of the neonatal animals. To gain additional biological insights, we profiled plasma levels of 736 metabolites at various time points to pinpoint the potential mechanisms behind the microbial genetic impact on the neonatal oxen.

## Methods

### Animals

In this study, 36 newborn oxen (Holstein) were randomly assigned to three groups and followed for 2 months after birth in Baoding, Hebei province, China. The groups included a control (CON) group, a rumen microbiota transplantation (RMT) group, and a rumen fluid transplantation (RFT) group. The newborn oxen were trained to feed milk using a bucket and then transferred to individual calf hutches. Starter (Supplementary Table S1) was provided *ad libitum* 3 days after birth and once daily in the morning thereafter. Pasteurized whole milk was fed twice daily at 0800 and 1800 h using a bucket, and the oxen were weaned 56 days after birth. RMT and RFT were performed by veterinarians, where the ruminal fluid used in RMT and RFT was collected from a healthy oxen (4-year-old, 600 kg, in the dry period) with a permanent rumen cannula 2 h after the morning feed. Fresh ruminal fluid was mixed with raw milk and fed to the oxen in the RMT group immediately after collection. For the RFT group, the ruminal fluid was autoclaved at 121°C and 15 psi for 15 min before feeding. A volume of 50 , 80 , and 110 ml of ruminal fluid was fed from Day 7 to Day 11, Day 21 to Day 25, and Day 42 to Day 46, respectively. Fecal and blood samples were collected at 15, 35, and 56 days after birth (Supplementary Fig. S1).

### Blood biomarkers

Blood samples were taken before morning feeding. A tube with ethylenediamine tetraacetic acid (EDTA) as an anticoagulant was used for isolating plasma, while a blank tube was used to separate serum. Plasma samples were used to analyze the concentrations of blood urea nitrogen, glucose, total cholesterol, and triglycerides, while serum samples were used to analyze the concentrations of total protein, albumin, alkaline phosphatase, aspartate aminotransferase, alanine aminotransferase, total antioxidant capacity, and malondialdehyde. The concentrations of blood biomarkers were analyzed using commercial kits from Nanjing Jiancheng Bioengineering Institute (Nanjing, China).

For the un-targeted metabolome analysis, plasma samples were resuspended with prechilled 80% methanol. The samples were incubated on ice for 5 min and centrifuged at 15 000 *g*, 4°C for 20 min. The resulting supernatant was injected into a ThermoFisher Vanquish UHPLC system coupled with an Orbitrap Q ExactiveTMHF mass spectrometer. Raw data files generated by ultra-high-performance liquid chromatography (UHPLC–MS/MS) were processed using Compound Discoverer 3.1 (CD3.1, ThermoFisher) to perform peak alignment, peak picking, and quantitation for each metabolite. The normalized data were used to predict the molecular formula based on additive ions, molecular ion peaks, and fragment ions. Peaks were then matched with the mzCloud (https://www.mzcloud.org/), mzVault, and MassList databases to obtain accurate qualitative and relative quantitative results. Metabolite annotations were performed using the KEGG database (https://www.genome.jp/kegg/pathway.html), HMDB database (https://hmdb.ca/metabolites), and LIPIDMaps database (http://www.lipidmaps.org/).

### Digestibility

Feed digestibility was determined using acid detergent insoluble ash as an internal marker [20]. Briefly, fecal, starter, and milk samples were collected from Day 13 to Day 15, Day 33 to Day 35, and Day 54 to Day 56, and then, the samples from each calf were pooled, dried at 55°C for 48 h, and then ground through a 1-mm screen for further analyses. The contents of dry matter (method 930.15) and crude protein (method 996.11) in the starter, milk, and fecal samples were determined according to AOAC International. The contents of neutral detergent fiber (NDF) and acid detergent fiber (ADF) in the starter and feces were measured using heat stable α-amylase and sodium sulfite [21]. The apparent total tract digestibility of starter was estimated as described previously [22]. The fecal score was monitored and recorded once daily after morning feed on every calf, using a 4-level scoring system [23].

### Metagenomic data generation and preprocessing

Fecal samples were collected via rectal swab from newborn oxen and promptly placed in a freezer (−20°C) within 15 min of production. The samples were then transported to the laboratory on dry ice and stored at −80°C until further processing. Fecal DNA was isolated using the QIAamp Fast DNA Stool Mini Kit (Qiagen, cat.51604), and sequencing was performed using a NovaSeq 6000 system (Illumina) The sequencing facility discarded low-quality reads from the raw metagenomic sequencing data, and Bowtie2 (v.2.1.0) was used to remove contamination reads [24, 25]. After filtering, an average of 36.8 million (s.d. ±3.6 million) paired reads per sample were obtained for subsequent analysis.

### *De novo* assembly and binning

**Figure 1:**
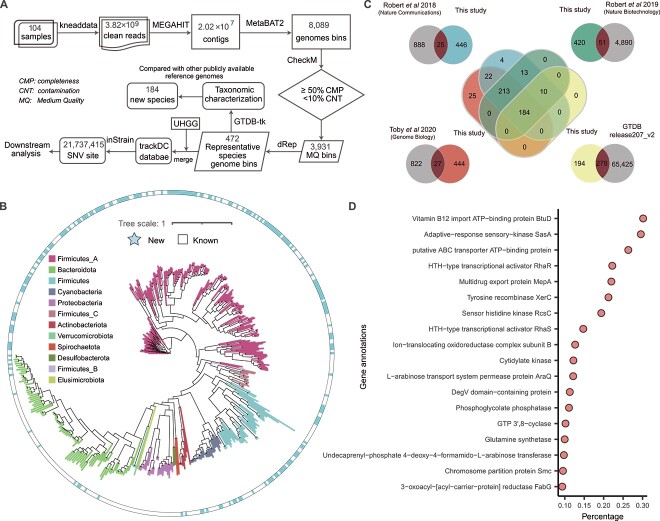
MAGs of the present study; (A) microbial genomic assembly workflow; (B) phylogenetic tree of 472 representative species-level MAGs; the colors on the branches represent the phyla to which the bacteria belong, and the blue color on the outer circle indicates that this is a newly identified species compared with existing genomic references; (C) comparison of 472 representative mags with publicly available reference genomes from the three recent studies; (D) functional annotation of microbial genes in the 184 newly identified MAGs; gene products are predicted based on UniProt, RefSeq, Pfam, and TIGRFAMs databases.

To reconstruct metagenome-assembled genomes (MAGs) from the fecal samples, a bioinformatics pipeline was used (Fig. 1A). After removing low-quality and contamination reads, adapter trimming was performed using Trimmomatic (v.0.33) [26], and the resulting clean reads were used as input for MEGAHIT (v.1.2.9) [27]. This resulted in 20 149 780 contigs longer than 200 bp from a total of 2 673 254 051 reads. Reads were then mapped back to the filtered assembly using Bowtie2 (v.2.1.0), and the resulting BAM files were converted to .bam format using SAMtools (v.1.15) [28]. Coverage was calculated using the jgi_summarize_bam_contig_depths script from the MetaBAT2 (v.2.12.1) package [29], and contig binning was performed using MetaBAT2 with contigs shorter than 1.5 kb discarded.

### Genome quality

After binning, we used CheckM (v.1.1.3) [30], which is based on the copy number of lineage-specific single-copy genes, to estimate the quality (completeness and contamination) using the “lineage_wf” workflow. We selected only genomes that passed the following criteria: ≥50% genome completeness and < 10% contamination. This also meets the “medium-quality draft” criteria according to recent guidelines [31]. After filtering through this process, a total of 3931 reconstructed genomes were identified, and the quality statistics, which were measured by analyzing single-copy core genes, are shown in Supplementary Table S2.

### Species-level representative metagenomic-assembled genomes

To cluster the total set of 3931 genomes at an estimated species level, we used dRep (v.3.2.2) [32]. We extracted the MAGs displaying the best quality and representing individual metagenomic species. dRep was run with options −pa 0.9 (primary cluster at 90%), −sa 0.95 (secondary cluster at 95%), −cm larger (coverage method: larger), and −con 10 (contamination threshold of 10%). The 95% threshold is commonly used for species-level clustering [33]. Genomes were scored based on their completeness, contamination, genome size, and contig N50, with only the highest scoring MAG from each secondary cluster being retained as the winning genome in the dereplicated set. Finally, we selected 472 species-level representative MAGs.

### Taxonomic classification and phylogenetic analyses

Taxonomic annotation of each reconstructed genome was performed using GTDB-Tk (v.2.0.0, database release 207) [34, 35] and the “classify_wf” function with default parameters. GTDB-Tk proposes bacterial taxonomy through the concatenation of 120 ubiquitous single-copy proteins, and the producing sequence alignments were used to generate a maximum-likelihood tree. The tree was visualized and annotated using Interactive Tree Of Life (v.4.4.2) [36].

### Gene prediction

The coding sequences (CDSs) for each of the 3931 MAGs were predicted and annotated using Prokka (v1.13.3) [37], which utilizes Prodigal (v2.6.3) [38]. Gene products are predicted based on UniProt, RefSeq, Pfam, and TIGRFAMs databases.

### Single nucleotide variant calling

To improve accuracy and comprehensiveness, we combined our study-specific genomes and a public genome database (Unified Human Gastrointestinal Genome, UHGG) to build the reference genomes. First, we merged all 472 species-level genomes and the entire UHGG genome collection, which contains all microbial species known to exist in the human gut so far, into a single FASTA file and created a Bowtie2 mapping index from it. The resulting FASTA file contained all the genomes we wanted to profile. Second, we created a scaffold-to-bin file that lists the genome assignment of each scaffold to show which scaffolds came from which genomes using the parse_stb.py script that comes with the program dRep (v.3.2.2). Subsequently, we mapped our metagenomic reads to the well-built reference database using Bowtie2 (v.2.1.0) to create BAM files. Next, we predicted genes for each genome using Prodigal (v.2.6.3) to create a genes file for gene-level profiling. Once all the necessary files were prepared, we called single nucleotide variants (SNVs) by running inStrain profile (v.1.3.2) with default parameters for each sample. We only selected SNVs whose “class” type is “SNV” as defined by inStrain and combined the scaffold, position, and reference base into a single site.

### Genomic dissimilarity

To compare the genomic differences between samples, we defined a genomic distance based on SNVs. First, we extracted the SNV sites (con_base) from each sample result file to obtain information about the reference base and its corresponding mutation type. We only considered the base that was supported by the most reads (con_base). Then, we merged all samples into a matrix where each row represented a SNV and each column represented the mutation type for each sample. We treated each column as a single sequence and aligned them to generate a phylogenetic distance matrix that contained the pairwise nucleotide substitution rate between samples using the Kimura 2-parameter method from the EMBOSS package [39]. To identify distinct strain clusters within species, the SNP haplotype distance matrix was normalized by dividing the maximal distance, and hierarchical clustering was performed using the complete method from the R basic function hcluster. Kruskal test was used to access the genomic dissimilates.

### Protein 3D structure and functional prediction

We used the AlphaFold2 artificial intelligence algorithm through ColabFold [40] and MMseqs2 [41] to model the protein structures based on multiple sequence alignments. Next, we predicted the functions of these proteins using DeepFRI [42], which is a Graph Convolutional Network that leverages sequence features from a protein language model and protein structures to predict protein functions.

### Association analysis

For each base site, there are four possible scenarios (i.e. A, T, C, G) for all samples regardless of the reference base. Initially, we excluded sites where a single base represented more than 90% of the population, and then we filtered out SNV sites that were present in <20% of the samples. Next, we only considered cases where two bases existed, which accounted for 97.36% of the data, and treated it as a binary variable. We then used linear and logistic regression models for continuous and binary traits, respectively, to establish microbial SNV associations with host phenotypes and metabolites. The formula used was: Metabolite/Phenotype ~ SNV + day. To identify the effect of RMT on SNV, we used a logistic model with the following formula: SNV ~ group + day. The false discovery rate (FDR) was calculated by using the Benjamini–Hochberg method [43].

## Results

### Genomic assembly resulted in 184 new species-level genomes

The study aimed to investigate the temporal dynamics of gut microbial strains at a single nucleotide resolution and the influence of early microbial intervention on the health and growth performance of neonatal oxen. For this, 36 neonatal oxen were randomly assigned to three groups: a CON group, a RMT group, and an autoclaved RFT group. Fecal samples were collected at 15, 35, and 56 days after birth and subjected to metagenomic sequencing. On average, we obtained 36.8 million paired reads for 104 fecal samples collected from newborn oxen.

To reconstruct microbial genomes in the gut of neonatal oxen, we carried out *de novo* assembly. To maximize the quantity of reconstructed genomes, we followed a single-sample assembly strategy as suggested in a recent study [44]. Using this pipeline (Fig. 1A), we obtained a total of 3931 MAGs that met the minimum information about a metagenome-assembled genome standard [31], with a completeness of ≥50% and < 10% contamination. The assembled genomes were of high quality, with an average completeness of 84% and a contamination rate of 1.7% (Supplementary Table S2).

We next checked the taxonomy of those MAGs by clustering all 3931 genomes with an average nucleotide identity (ANI) threshold of 95%, resulting in 472 representative prokaryotic species (Supplementary Table S3). According to the taxonomic classifications using GTDB-Tk [34], these genomes mainly belong to the phyla *Firmicutes* and *Bacteroidota* (Fig.1B, Supplementary Table S3). We compared these representative genomes with existing microbial genomes obtained from cattle, including RUG [45], RUG2.0 [46], and African MAGs [47]. At 95% ANI, we found that 446, 420, and 444 of them were novel when compared with RUG, RUG2.0, and African MAGs, respectively (Fig. 1C**)**. Overall, 397 out of the 472 representative genomes were not covered in the above-mentioned studies (Fig. 1C). Furthermore, we mapped the 397 unique species-level representative MAGs using GTDB-Tk. It was found that 184 of these MAGs could not be taxonomically classified at the species level, 23 could not be annotated at genus level.

To investigate the functionalities of these novel species, we used Prokka [37] to annotate the functional genes in the newly discovered genomes. Our analysis revealed that these novel species have variable functionalities, ranging from adaptive-response sensory-kinase to glutamine synthetase, while a substantial number of genes were not annotatable, with 47% of the genes being hypothetical (Fig. 1D, Supplementary Table S4).

In summary, the newly constructed genomes are a valuable supplement to existing resources for microbiome research in neonatal oxen, providing additional insight into the taxonomy and functional capabilities of gut microbes in this population.

### Temporal variability of the gut microbial strains in neonatal oxen

Investigating the temporal colonization of gut microbial strains in neonatal oxen is crucial as it can provide insights into the mechanisms that drive the development of oxen and identify potential targets for interventions to promote host health. Here, by comparing the number of MAGs obtained from each calf at different time points, we found that the number of MAGs obtained in neonatal oxen increases over time (Supplementary Fig. S2A). Moreover, this increase was not biased by differences in sequencing depth as significant negative but not positive correlations were observed (Supplementary Fig. S2B), demonstrating a rapid influx of gut microbial strains after birth during the first few weeks of neonatal calf development.

To study the temporal colonization of microbial strains in the gut of neonatal oxen, we utilized shotgun metagenomic data to conduct strain-level analysis using SNVs. To achieve this, we combined our MAGs with the UHGG [48] database, which is currently the most comprehensive microbiome sequence resource available. This enabled us to not only investigate study-specific microbial strains but also other existing strains. We aligned sequencing reads to this customized reference database and performed SNV calling using the inStrain [49] algorithms.

**Figure 2:**
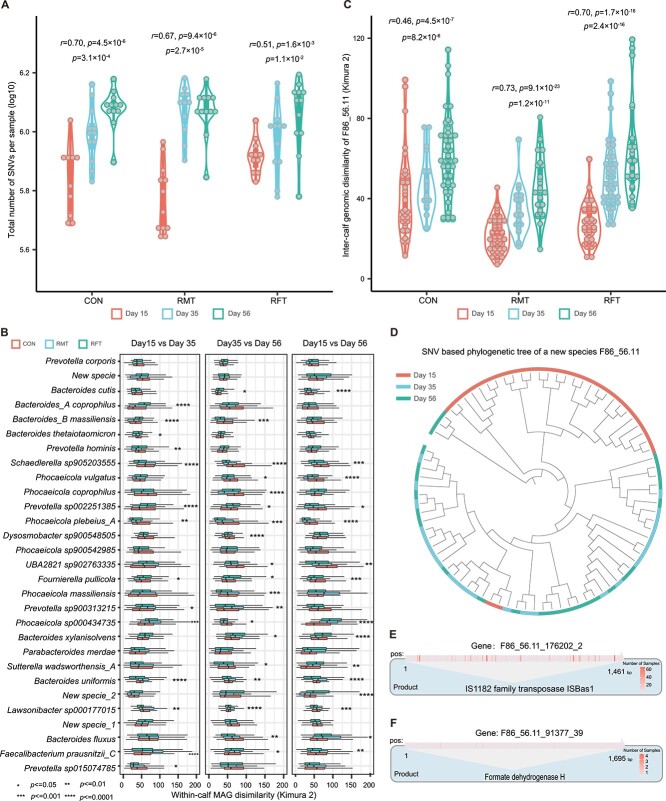
Temporal variability of gut microbial strains in neonatal oxen; (A) the total number of SNVs detected per sample; each dot represents one sample; the Spearman and Kruskal tests are used to assess the temporal correlation and within-group differences of SNVs obtained per sample; (B) the temporal genomic dissimilarity of species-level MAGs is presented; within-group genomic distance is calculated using the Kimura 2-parameter method; (C) the inter-calf genomic dissimilarity of a novel species-level MAG *F86_56.11*; the Kruskal test and the Spearman correlation are used to assess temporal differences and associations within the group; (D) phylogenetic tree of the novel species *F86_56.11*; colors represent the time of sampling; (E) SNVs detected within the genes encoding family transposase are illustrated using a heatmap; gene length and the number of samples with SNVs are shown; (F) SNVs detected within the genes encoding FDH are illustrated using a heatmap; gene length and the number of samples with SNVs are shown; CON: water; RMT: ruminal fluid; RFT: autoclaved ruminal fluid.

In total, 21 737 415 unique SNVs were detected in the 104 samples, derived from 1209 representative MAGs in our customized reference database, with a range of 440 250 to 1 564 434 SNVs per sample. Similar to the number of assembled MAGs, we observed an increase in the number of SNVs over time across all three groups (Fig. 2A), indicating an increase in genomic diversity of the gut microbiome in newborn oxen with time. The bacterial species with the highest number of SNVs included *Bacteroides uniformis*, *Parabacteroides faecavium*, and *Phocaeicola vulgatus*. Upon further analysis of within-calf genetic differences in SNVs, we identified MAGs that exhibited significant temporal changes in their SNVs, such as *Alistipes senegalensis*, *Prevotella sp002353825*, and *Bacteroides gallinarum*, while *Roseburia inulinivorans*, *Bacteroides intestinalis*, and *Enterenecus faecium* showed relatively low genetic variability (Fig. 2B, Supplementary Table S5).

Interestingly, we found that the genetic variability of a novel species, *F86_56.11*, varied substantially over time. The dissimilarities between oxen based on SNVs of this species increased with time and were consistently the lowest on Day 15 (Kruskal test, *P* < 1.0 × 10^−5^, Fig. 2C). Phylogenetic analysis further showed that the species had two distinct strains (Fig. 2D), with a significant enrichment of Strain 1 observed on Day 15 compared to Day 35 and Day 56 (Fisher exact test, *P* < 1.07 × 10^−10^, Fig. 2D), indicating the high temporal variability of this bacterium.

To investigate the potential mechanisms responsible for the temporal genetic variability observed in *F86_56.11*, we analyzed genes with SNVs that displayed significant temporal differences. Our genomic annotation analysis revealed that several genes that encode family transposase exhibited significant temporal variability in *F86_56.11* (Kruskal test, *P* < 0.05, Fig. 2E, Supplementary Table S6). Transposase is a protein that facilitates the movement of transposons to different locations within the genome. It can bind to transposons and promote their movement within the genome, driving genome mutation [50]. The activity of transposable elements and their associated transposases can significantly impact the evolution and adaptation of bacteria to their environment [50].

In addition, we found that SNVs in a 1695 bp *fdhF_2* gene that encodes formate dehydrogenase H (FDH) also exhibited temporal differences (Kruskal test, *P* = 2.5 × 10^−2^, Fig. 2F, Supplementary Table S6). FDH is a crucial enzyme involved in host immunity-related formate metabolism [51], a key intermediate in the production of short-chain fatty acids (SCFAs) [52]. The temporal changes in FDH could be related to fiber digestion in neonatal oxen because ADF and NDF also exhibited significant changes over time (Kruskal test, *P* < 9.6 × 10^−4^, Supplementary Fig. S3). These results suggest that the observed temporal genetic variability in microbial strains in neonatal oxen could be due to selective pressure on the microbes, which drives them to evolve and adapt to changing conditions in the gut environment. This adaptive process could involve potential mechanisms underlying the temporal changes in transposable elements and the metabolism of SCFAs, such as FDH.

### Microbial transplantation alters the gut microbial strains in neonatal oxen

**Figure 3:**
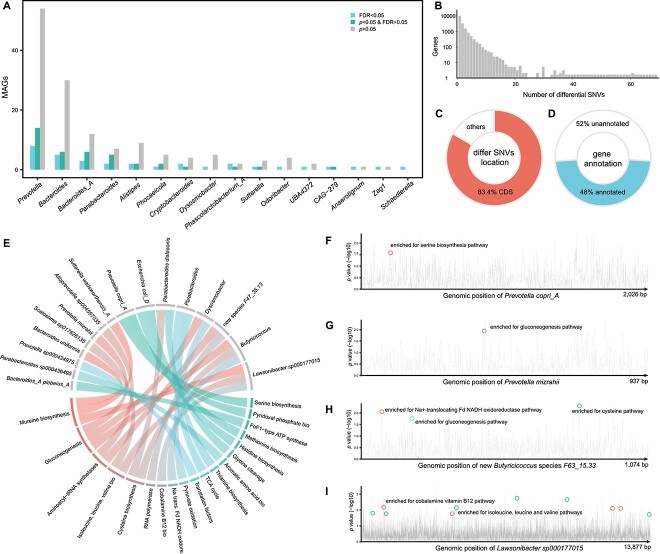
MT alters the gut microbial strains in neonatal oxen; (A) MAGs show differences in their genetic makeup between groups; the X-axis represents different genera, while the Y-axis represents the number of MAGs; the blue bars represent MAGs that showed significant differences at FDR < 0.05; (B) the distribution of microbial genes with differential SNVs between groups; the X-axis represents the number of differential SNVs within a gene, while the Y-axis represents the number of microbial genes; (C) the location of differential SNVs within genes; (D) the percentage of microbial genes that can be functionally annotated; (E) an overview of 26 significant functional enrichments based on microbial genes with differential SNVs per MAG; (F–I) differential genomic loci altered by MT and the functional pathway enriched by genes with differential SNVs in *P. copri_A*, *P. mizrahii*, a new *Butyricicoccus species* (*F63_15.33*), and *Lawsonibacter sp000177015*; the X-axis represents the genomic position of SNVs, while the Y-axis displays the P-values (−log10) of differential SNVs between groups.

In addition to examining the temporal shifts of gut microbial strains in neonatal oxen, we also investigated whether ruminal microbiota transplantation could alter the colonization of specific microbial strains in the gut of these oxen. To do this, we compared the genomic dissimilarity (Kimura 2-parameter distance) of 686 MAGs that were present in all three groups, and significant differences were found for 33 MAGs (Kruskal test, FDR < 0.05, Supplementary Table S7). Notably, many of the MAGs that were significantly altered by RMT were from *Prevotella* and *Bacteroides* species, such as *Prevotella mizrahii*, *Prevotella sp900543585*, *Bacteroides caccae*, and *Bacteroides fragilis* (Fig. 3A, Supplementary Fig. S4, Supplementary Table S7).

To further investigate the functional differences underlying the differential MAGs, we analyzed genes with differential SNVs between the groups. In total, we identified 16 497 genes with at least one differential SNV between the groups (Supplementary Table S8), and 3766 of them (accounting for 22.8% of total genes) had multiple differential SNVs (more than 3, Fig. 3B). In total, 83.4% of differential SNVs were located within the CDS of genes (Fig. 3C, Supplementary Table S8), and 7914 out of 16 497 genes (48.0%) were functionally annotated (Fig. 3D, Supplementary Table S8). These genes encoded a wide range of bioactivities that may have been induced by RMT, including the metabolism of SCFAs, vitamins, and amino acids (Supplementary Table S8). All of these are important for neonatal calf growth and development, suggesting that early microbial interventions have a significant effect on reshaping the genetic makeup and functionalities of the gut microbiome in neonatal oxen.

As we have the clusters of orthologous genes (COGs) [53] ids for genes with differential SNVs (Supplementary Table S8), we customized the COG pathway database [53] to calculate pathway enrichment based on genes with differential SNVs per MAG. In total, we identified 26 significant enrichments between 19 microbial pathways and 16 MAGs (Fisher’s exact test, FDR < 0.05, Fig. 3E, Supplementary Table S9). For example, *Prevotella copri_A* and *P. mizrahii* were enriched for antioxidant-related serine pathways (*P* = 1.4 × 10^−2^, Fig. 3F) and SCFA-related gluconeogenesis pathways (*P* = 1.7 × 10^−2^, Fig. 3G). Moreover, we observed that genes with differential SNVs in a new *Butyricicoccus* species (F63_15.33) were mainly enriched for SCFA-related pathways (*p*_gluconeogenesis_ = 2.5 × 10^−2^, *p*_Na + −translocating Fd_NADH oxidoreductase_ = 2.5 × 10^−2^), as well as antioxidant-related cysteine biosynthesis pathways (*P* = 3.2 × 10^−2^, Fig. 3H). Additionally, *Lawsonibacter sp000177015* was enriched for cobalamin (vitamin B12, *P* = 1.2 × 10^−5^, Fig. 3I) and branched-chain amino acid biosynthesis pathways, including isoleucine, leucine, and valine (*P* = 3.0 × 10^−3^, Fig. 3I). These results further emphasize the importance of microbial interventions during the early days of life in neonatal oxen to reshape their gut microbial strains and modulate their metabolism for better health and growth.

### Microbial single nucleotide variations associated with plasma metabolites in newborn oxen

We characterized significant temporal and RMT-induced differences in the genetic makeup of microbial strains. However, a considerable number of SNVs cannot be functionally annotated (Fig. 3D). To gain a deeper understanding of how SNVs might drive host pathophysiology, we hypothesized that metabolites play a crucial role in host–microbe interactions. Therefore, the associations between SNVs and plasma metabolites were assessed. We began by selecting SNVs present in more than 20% of the samples and with a minor allele frequency of 10%. In such a way, 787 964 SNVs were finally associated with 736 untargeted plasma metabolites to identify potential microbial genetic determinants of plasma metabolites in neonatal oxen.

**Figure 4:**
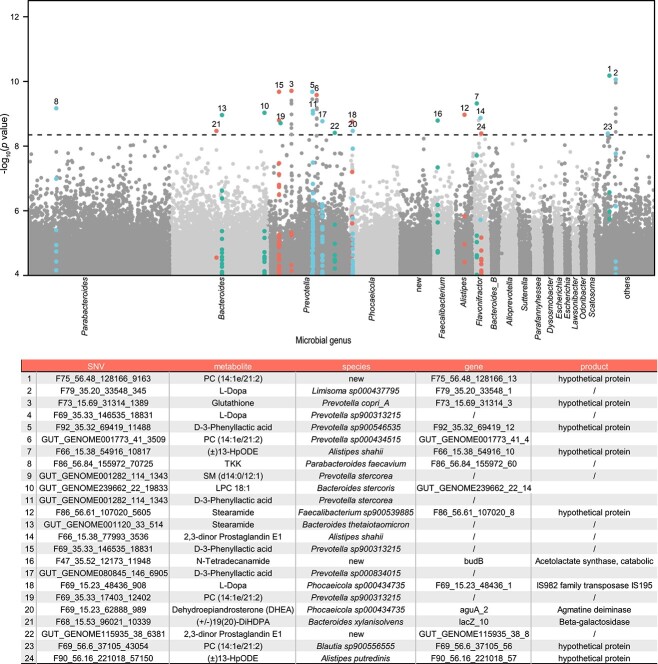
Microbial genetic determinates of plasma metabolites; the Manhattan plot shows the association between microbial SNVs and 736 untargeted plasma metabolites; each point in the plot represents a genetic locus, with the X-axis indicating the genomic position of MAGs in different genera and the Y-axis representing P-values of linear associations; the dashed line represents the study-wise significant line (*P* < 4.4 × 10^−9^), and detailed information about the associations is provided in the table; for SNVs with metabolite association above the study-wise significant line, their associations with other metabolites are also highlighted with the same colors even below the study-wise significant line.

A total of 52 significant associations were identified between 50 SNVs from 19 MAGs and 13 metabolites (Supplementary Table S10), with an FDR of <0.05 and corresponding *P*-values <4.4 × 10^−9^. Highly correlated SNVs (*R*^2^ > 0.9, Supplementary Fig. S5) from the same MAGs associated with the same metabolite were filtered, resulting in 24 independent SNV-metabolite associations (Fig. 4). The most significant association was found between a SNV from a new species (*F75_56.48*) and the plasma level of phosphatidylcholine (*P* = 6.63 × 10^−11^). Among the 24 associations, eight were observed for SNVs from the genus *Prevotella*, with metabolites mainly including glutathione, D-3-phenyllactic acid (PLA), and L-dopa. Glutathione has been reported to affect virulence and bacterial pathogenesis, and the host may use glutathione to modulate its response against bacterial incursions [54]. PLA is capable of inhibiting the growth of many microorganisms [55]. Additionally, an SNV in the gene coding agmatine deiminase was associated with dehydroepiandrosterone (DHEA), an important endogenous androgen steroid hormone [56]. Agmatine deiminase [57] is involved in the microbial putrescine biosynthesis pathway, which is related to reproductive processes such as spermatogenesis, sperm motility, follicular development, and ovulation [58].

The associations observed between unannotated SNVs and metabolites provide a valuable resource for gaining a deeper understanding of the mechanisms behind host–microbe interactions in neonatal oxen. These associations could potentially guide targeted mechanistic investigations to determine the impact of variable microbial strains on the health and growth of neonatal oxen. By identifying specific SNVs that are associated with a particular metabolite, we may be able to unravel the complex interplay between the microbial genome and host physiology. Ultimately, this information may lead to the development of new interventions and treatments to improve the health and growth of neonatal oxen.

### Microbial single nucleotide variations-related metabolites associated with phenotypes of neonatal oxen

**Figure 5:**
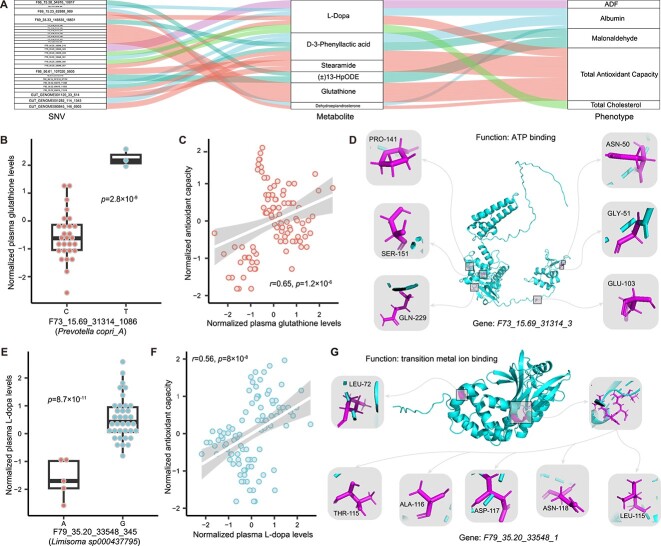
Microbial SNVs-related metabolites associated with phenotypes of neonatal oxen; the Sankey diagram shows the association between SNVs, metabolites, and phenotypes of newborn oxen; (B) the association between locus F73_15.69_31314_1086 of *P. copri_A* and the levels of plasma glutathione; (C) the correlation between plasma glutathione levels and the antioxidant capacity of neonatal oxen; the X-axis represents the normalized plasma glutathione levels, while the Y-axis represents the normalized antioxidant capacity of neonatal oxen; the fitted linear regression line is shown with a 95% confidence interval; the Spearman correlation coefficient and P-value are shown; (D) the putative protein structure of gene F73_15.69_31314_3 and the mutation sites on this gene; (E) the association between different base types at locus F79_35.20_33548_357 of *Limisoma sp000437795* and the levels of plasma L-Dopa; (F) the correlation between plasma L-Dopa levels and the antioxidant capacity of neonatal oxen; the X-axis represents the normalized plasma L-Dopa levels, while the Y-axis represents the normalized antioxidant capacity of neonatal oxen; the fitted linear regression line is shown, with the Spearman correlation coefficient and P-value; (G) the putative protein structure of gene F79_35.20_33548_1 and the mutation sites on this gene.

To further understand the relationships between SNV-related metabolites and neonatal calf phenotypes, we performed an association analysis between metabolites and phenotypes. Of the 13 metabolites associated with 50 SNVs (FDR < 0.05, Supplementary Table S10) and the 147 metabolites associated with 14 phenotypes (FDR < 0.05, Supplementary Tables S11 and S12), we found that 6 metabolites were associated with both (Fig. 5A, Supplementary Table S13). The phenotypes involved in these associations included plasma total cholesterol, albumin, malondialdehyde, total antioxidant capacity, as well as the digestibility of ADF (Fig. 5A).

Most SNV-related metabolites were associated with the total antioxidant capacity of neonatal oxen, including L-dopa, PLA, stearamide, glutathione, and DHEA. The strongest associations were observed between glutathione and SNV sites at the gene *F73_15.69_31314_3*. For instance, oxen with a C or T base at loci F73_15.69_31314_1086 had different levels of plasma glutathione (*P* = 2.8 × 10^−9^, Fig. 5B). Glutathione is an essential molecule for cellular homeostasis and defense against oxidative damage in various diseases [59], and robust correlation between plasma glutathione levels and the antioxidant capacity of neonatal oxen was observed (*r*_Spearman_ = 0.65, *P* = 1.2 × 10^−6^, Fig. 5C). However, the function of this gene based on Prokka was uncharacterized, we then applied AlphaFold2 [60] to predict the structure of the encoded protein (Fig. 5B), and the function was further annotated as ATP binding according to the protein structure estimated by DeepFRI [42]. Glutathione is formed by the sequential reaction of L-glutamic acid, L-cysteine, and glycine catalyzed by GSH I and GSH II in the presence of ATP [61]. It was suggested that this protein may bind ATP to influence the synthesis and transport of glutathione [62]. Thus, mutations in this gene may play an important role in the biosynthesis of glutathione via structural regulations (Fig. 5D).

We also found that multiple SNVs in *F79_35.20_33548_1* were associated with plasma L-dopa levels. Oxen with an A or G base at loci F79_35.20_33548_357 had different levels of L-dopa (*P* = 8.7 × 10^−11^, Fig. 5E), and we observed a significant correlation between plasma L-dopa levels and antioxidant capacity (*r*_Spearman_ = 0.56, *P* = 8.0 × 10^−8^, Fig. 5F). L-dopa is an important precursor for melanin biosynthesis, which is dependent on tyrosinase containing metal ions and plays a vital role in protecting cells from oxidative stress [63, 64]. The function encoded by *F79_35.20_33548_1* is the binding of metal ions, which can be regulated by SNVs through structural changes (Fig. 5G). Thus, mutations in multiple sites of the protein F79_35.20_33548_1 may impact the metabolic process of melanin and ultimately affect the antioxidant capacity. Those results provide putative mechanistic insights by identifying specific microbial genetics and functions and highlight which metabolites may be involved in the impact of the gut microbiome on the health of neonatal oxen.

## Discussion

It is important to understand how the gut microbiome affects the health of neonatal oxen by studying the temporal dynamics of the microbiome in early life. Previous studies using 16S rRNA gene sequencing have examined changes in the composition of the gastrointestinal microbiota in newborn oxen during the first few weeks after birth. Our study, which used metagenomic sequencing, provides a more detailed understanding of the genetic makeup of the microbiome over time, revealing that newborn oxen are rapidly colonized by microbial strains after birth and then undergo strong genetic selection in the first few weeks of life. We generated thousands of MAGs and identified 397 novel species-level MAGs from fecal samples of newborn oxen, which were not previously represented in existing rumen databases. This may be due to the differences between fecal and rumen samples, as well as regional differences. Our study was conducted in China, while other databases have focused on cattle from Scotland [45, 46] or Africa [47]. These newly constructed genomes provide additional resources for future microbiome research in *Bos taurus*.

We investigated the temporal colonization of microbial strains over time in newborn oxen by analyzing SNV profiles. We utilized a combination of our sample-specific genomes and the UHGG sequence resource to accurately analyze SNVs in both study-specific and existing microbial strains. Our results showed that the genetic stability of gut microbes varied substantially across different species. Some species, such as *R. inulinivorans*, *B. intestinalis* and *Enterococcus faecium*, showed relatively low temporal changes over time. Interestingly, previous studies in humans have also shown that some of these species, such as *Bacteroides* [65], are colonized in early life and exhibit high genetic stability in childhood [66]. Moreover, we used metagenomics sequencing to not only identify the strain SNV profiles but also examine the functional differences that may drive these SNV-based genetic clusters. We found that the temporal changes in genes involved in SCFA metabolism may be related to the changes in fiber digestion observed in neonatal oxen over time. Our study also demonstrates that metagenomic SNVs are an extra source of information to understand the role of the gut microbiome in neonatal animals. Based on SNVs profile, we found that the gut microbiome of newborn oxen can be altered by early microbial interventions at strain level. For instance, the genomic makeup of *Prevotella* and *Bacteroides* species was significantly altered.

In addition, our metagenome-wide microbial SNV association study on 736 plasma metabolites identified 24 independent SNV-metabolite associations. One-third of these associations were observed for SNVs from the genus *Prevotella* and the related metabolites, including glutathione, PLA, and L-dopa. Notably, a variety of metabolites, including stearamide, PLA, and glutathione were also associated with immune phenotypes of neonatal oxen. By identifying the genetic determinants of plasma metabolites in neonatal oxen, our study sheds light on the potential role of SNVs in driving host pathophysiology. Specifically, the significant associations between SNVs from microbial strains and plasma metabolites suggest that microbial genetic variation may play a crucial role in shaping host–microbe interactions and contribute to the regulation of host metabolism [67]. These findings highlight the importance of further investigating the molecular mechanisms underlying the observed associations and their potential implications for understanding the interplay between host and microbial genetic variation in health and disease.

The study presented in this paper provides valuable insights into the temporal dynamics of the gut microbiome in neonatal oxen and their potential linkages to their health and growth. However, there are several limitations that need to be acknowledged. First, there may rapid microbial colonization in newborns during the first week and intensive time points should be taken, and if possible, taking different locations of gastrointestinal tract into account might give extra insights instead of fecal samples. Second, we have characterized many novel microbial strains and have highlighted their importance in neonatal oxen. However, the ability to culture and isolate those strains is currently unknown, and further work is needed to determine their viability and potential for experimental validation. Third, it is important to note that associations observed in this study do not necessarily imply causation. Additional research is needed to investigate the mechanisms underlying the observed associations and their potential implications for improving early life gut microbiota in neonatal oxen. Despite these limitations, the study provides novel insights into the temporal dynamics of the gut microbiome in neonatal oxen and highlights the potential for future research to develop strategies for improving gut microbiota in early life.

## Conclusions

In summary, we present a longitudinal investigation of the temporal dynamics of the gut microbiome at the single nucleotide level, and the impact of early microbial interventions on neonatal oxen. We assembled gut microbial sequencing data from 104 samples of 36 neonatal oxen, resulting in a total of 3931 MAGs. Our dataset includes 472 unique species-level MAGs (95% ANI), of which 184 have not been previously reported in cattle, thereby providing an additional resource for microbiome research in *B. taurus*. We characterized the temporal dynamics of the gut microbiome at the SNV level and observed a rapid influx of microbes after birth, followed by strong selection during the first few weeks of life. Additionally, we found that microbial interventions can reshape the gut microbial strains of neonatal oxen. We also assessed the association between millions of microbial SNVs and hundreds of plasma metabolites, revealing the genetic regulation of the gut microbiome on host metabolism. Our results show that microbial genetic regulation on host metabolism can be linked to health status and growth performance of neonatal oxen.

## Supplementary Material

SupFigures_wrad022

SupTables_wrad022

## Data Availability

All relevant data supporting the key findings of this study are available within the article and its Supplementary Information files. The raw metagenomic sequencing data used for the analysis presented in this study are available from the European Nucleotide Archive (ENA) under accession id PRJEB42631. The metabolic profiles are available from the National Genomics Data Center (NGDC) with accession id OMIX003726. We summarized the characterized metabolic gene clusters (MGCs) based on the identified MAGs and uploaded the results to CNCB (https://www.cncb.ac.cn/) with accession id OMIX005086. The taxonomy summary statistics table was also submitted to CNCB with accession id OMIX005088. Analysis code is available via: https://github.com/MicrobiomeCardioMetaLab/trackDC.SNV_project.
