# Supplementary material for: Temporal colonization and metabolic regulation of the gut microbiome in neonatal oxen at single nucleotide resolution
Source: ISME J. 2024 Jan 10;18(1):wrad022. doi: 10.1093/ismejo/wrad022 (PMC10833086; doi:10.1093/ismejo/wrad022)

**Figure S1. Study design and overview of the trackDC.**


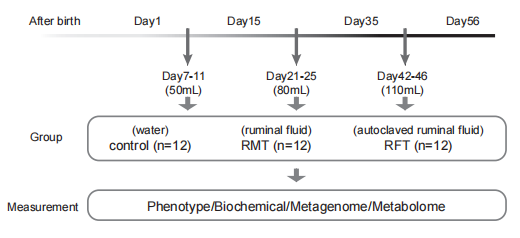


**Figure S2. Temporal differences in MAGs and sequencing depth. A**. Temporal changes of MAGs obtained per sample. Spearman and Kruskal tests are used to assess the temporal correlation and within-group differences of MAGs obtained per sample. **B**. Temporal changes of sequencing depth per sample. Spearman and Kruskal tests are used to assess the temporal correlation and within-group differences of sequencing reads obtained per sample. CON: water; RMT: ruminal fluid; RFT: autoclaved ruminal fluid.


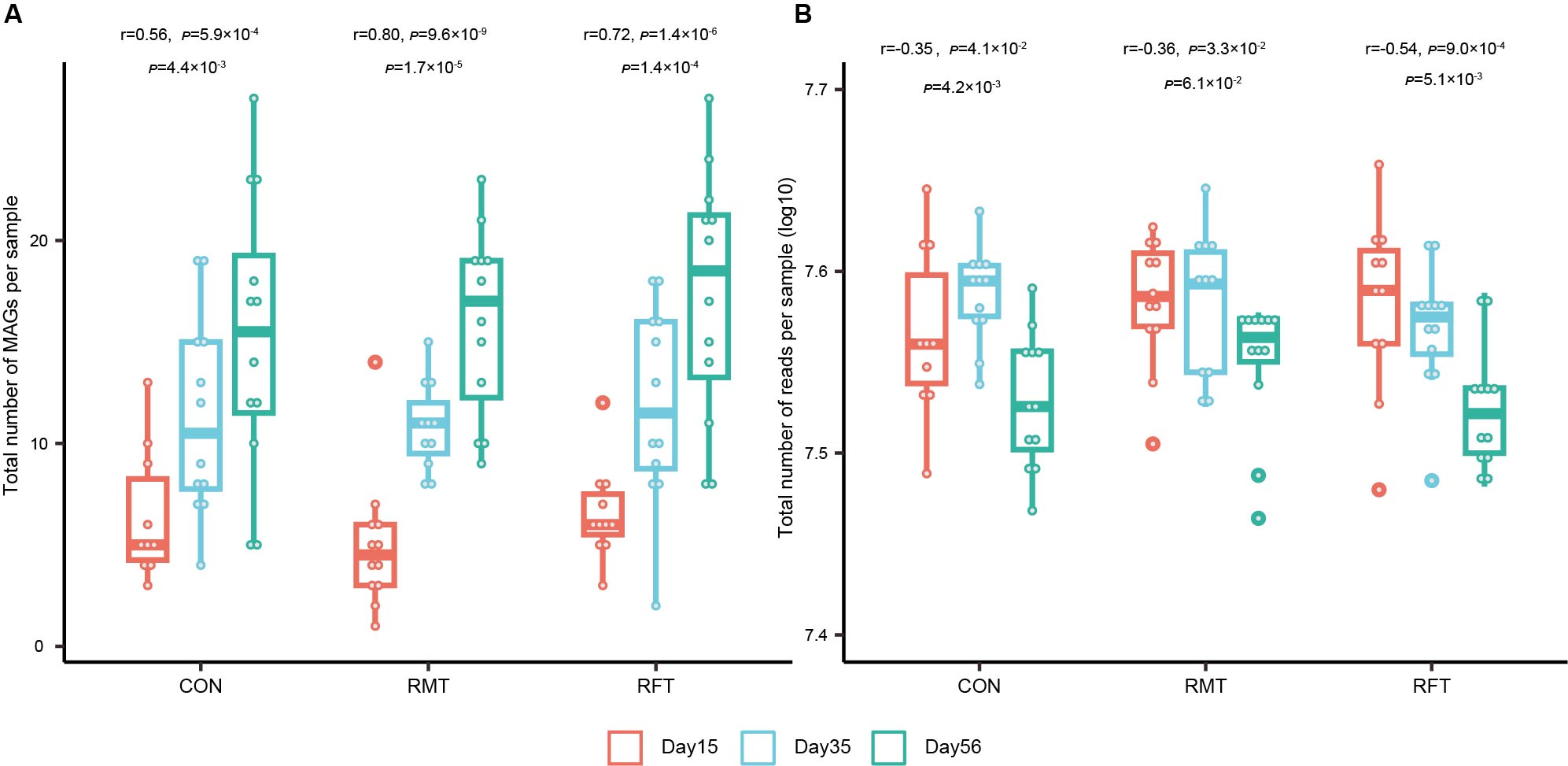


**Figure S3. Temporal changes of fiber digestibility in neonatal calves. A.** Temporal changes of neutral detergent fiber (NDF) digestibility. Each dot represents one sample. **B.** Temporal changes of acid detergent fiber (ADF) digestibility. The P-values from the Kruskal tests are shown. Each dot represents one sample.CON: water; RMT: ruminal fluid; RFT: autoclaved ruminal fluid.


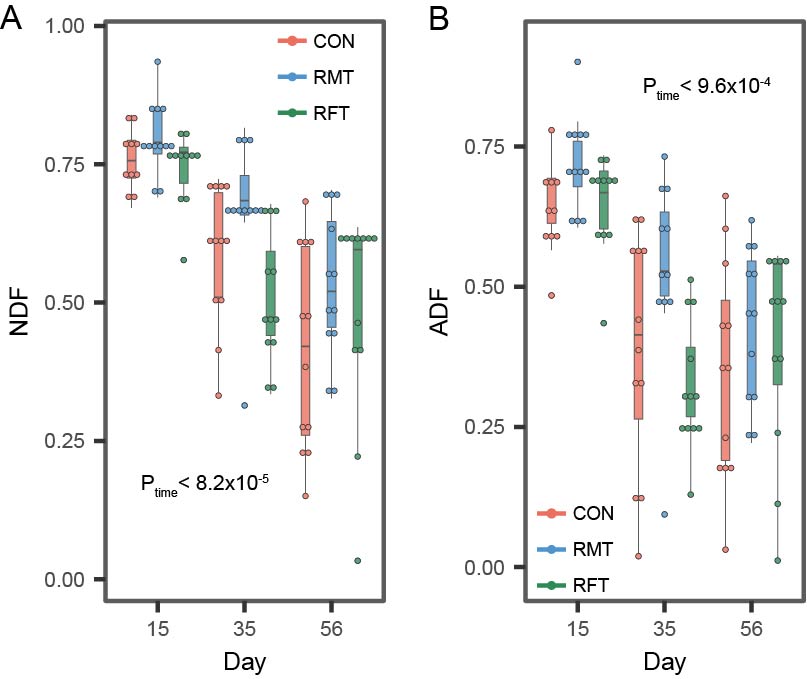


**Figure S4. Genomic dissimilarity of MAGs between groups.** Inter-calf MAG dissimilarities are calculated using the Kimura 2-parameter method. The Kruskal test is used to assess differences between groups. The comparison between each pair of groups was performed using the Wilcox test. CON: water; RMT: ruminal fluid; RFT: autoclaved ruminal fluid.

**
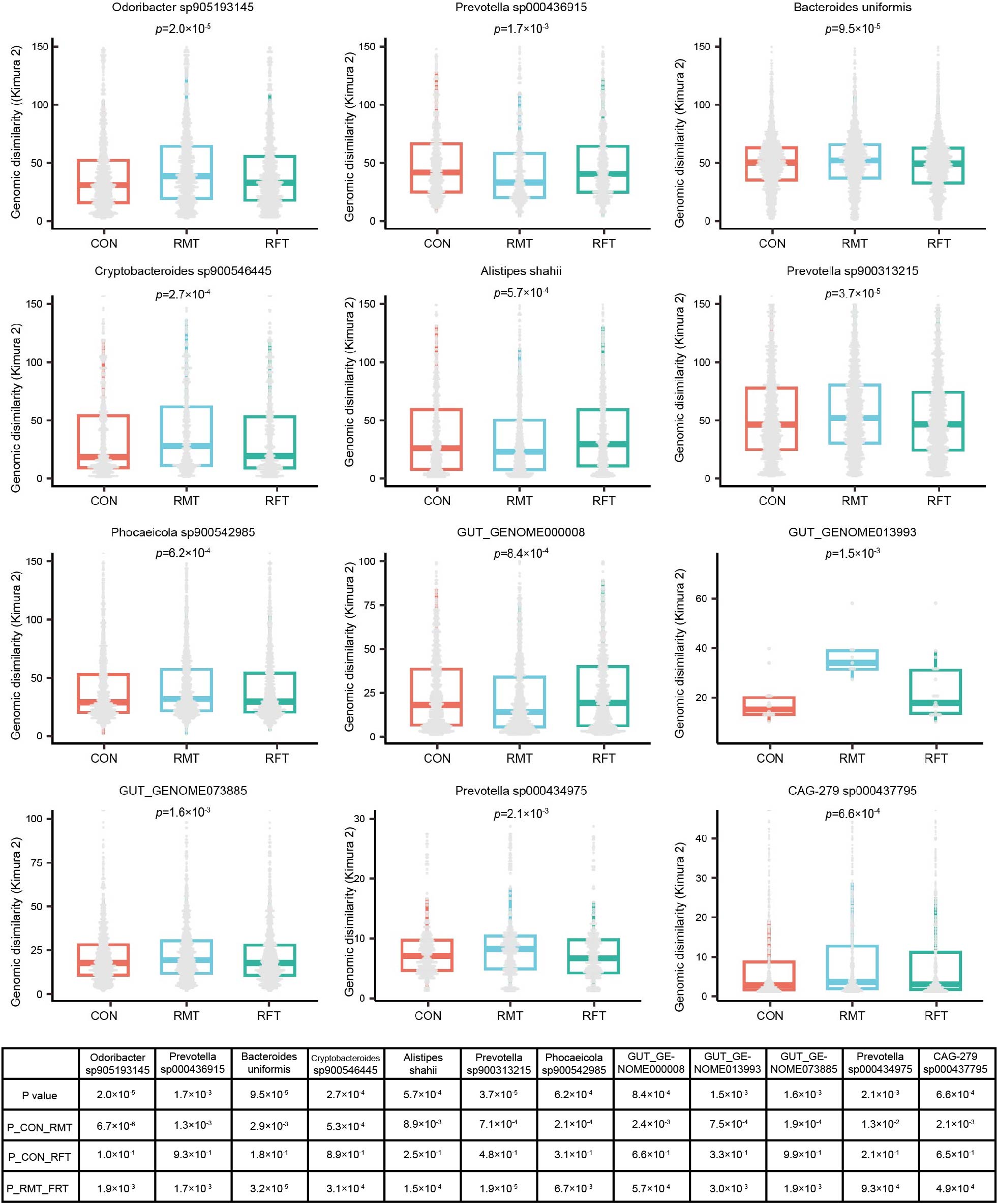
**

**Figure S5. Inter-correlations of microbial SNVs.** The heatmap shows Spearman correlations between SNVs with significant associations to plasma metabolites. The darkness of colors represent the correlation strength.


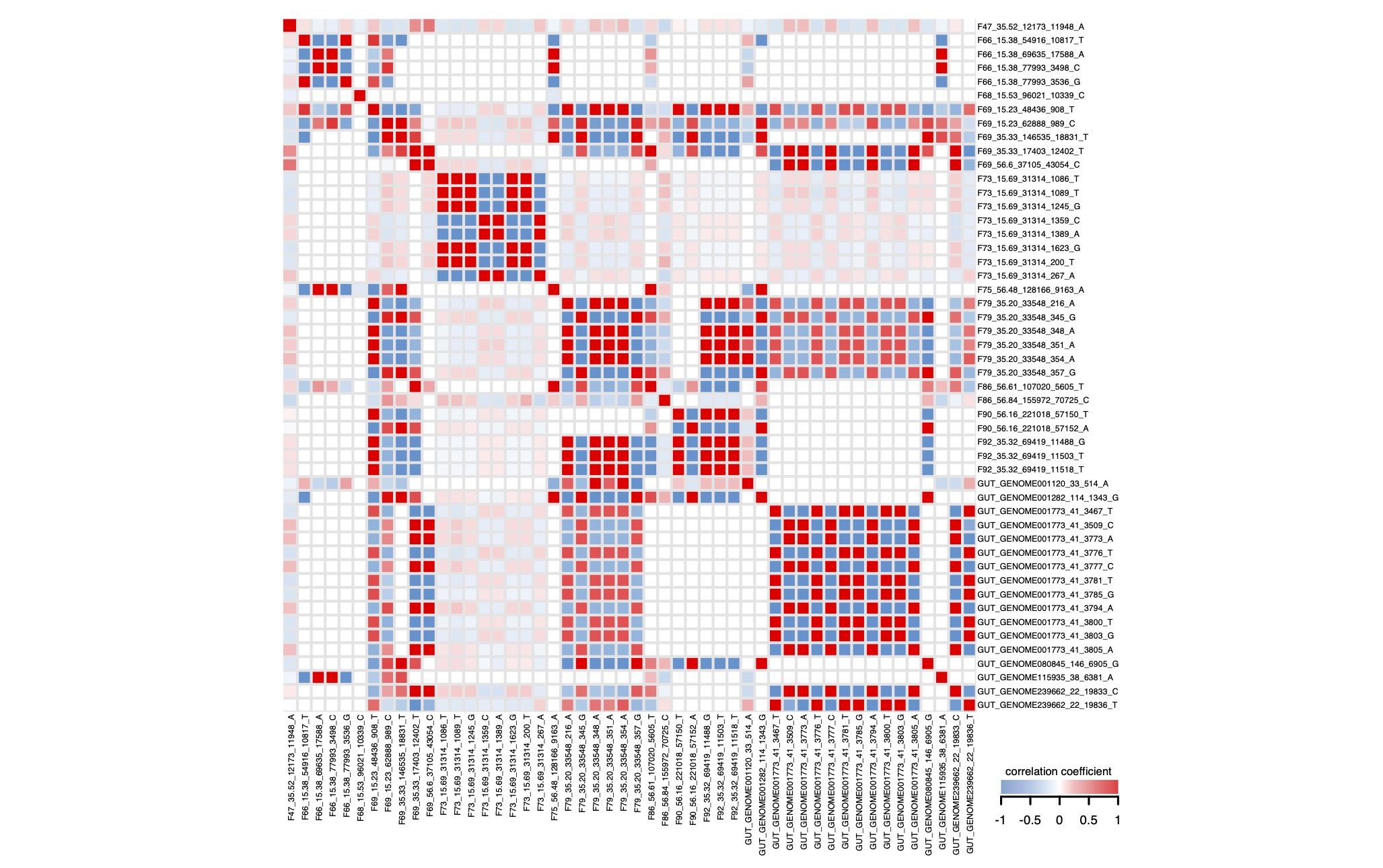

Supplement: SupFigures_wrad022 [file supfigures_wrad022.docx]
